# OP41 Using Horizon Scanning To Identify Emerging Quantum Technologies In Health Care

**DOI:** 10.1017/S0266462325100998

**Published:** 2025-12-29

**Authors:** Ross Fairbairn, Oshin Sharma, Imogen Forsythe, Elizabeth Green, Oleta Williams, Andrew Mkwashi

## Abstract

**Introduction:**

Quantum technologies, driven by principles of quantum mechanics like superposition and entanglement, have shown transformative potential in health and medicine. However, the understanding of these technologies in early to late stages of development is limited. This study aimed to investigate the emerging applications of quantum technologies in health care, with a focus on quantum computing, diagnostics, and therapeutics.

**Methods:**

Horizon scanning researchers, in collaboration with information specialists and with steering from subject matter experts at National Health Service England, developed search strategies for identifying relevant emerging and established quantum applications in healthcare domains such as drug design, personalized medicine, diagnosis assistance, and treatment or interventions. We conducted a comprehensive review of this landscape by analyzing data from clinical trials, published literature, and soft intelligence sources. Primary and secondary sources were reviewed systematically using a combination of traditional methods and the Innovation Observatory’s in-house semi-automated Search Companion for Advanced News Article Retrieval (SCANAR) tool to capture non-traditional soft intelligence sources.

**Results:**

The scan identified 116 quantum technologies in health care, with magnetoencephalography (MEG), quantum dots, and superconducting quantum interference devices (SQUIDs) being the most frequently used for brain mapping, imaging, and cardiac diagnostics. Over half (54%) of these technologies assist in diagnosis, while others are used in drug design (15%), treatment (15%), and precision medicine (13%). Quantum computing applications (28%) and quantum dots (24%) were the most common applications identified. Non-commercial trials dominated (88%), reflecting a focus on advancing care. Additionally, 27 percent of the technologies incorporate artificial intelligence, mainly for personalized medicine and imaging diagnosis.

**Conclusions:**

Quantum technologies in health care are advancing areas of diagnostics, personalized medicine, and therapeutics. Diagnostics are the most popular area, most frequently using technologies such as MEG, SQUIDs, and quantum dots. Quantum computing algorithms accelerate drug discovery and precision medicine. Although promising, integrating quantum technologies into health care faces challenges in infrastructure demands and regulatory frameworks, and requires professional training in quantum literacy.

